# Study protocol: developing, disseminating, and implementing a core outcome set for selective fetal growth restriction in monochorionic twin pregnancies

**DOI:** 10.1186/s13063-018-3153-y

**Published:** 2019-01-09

**Authors:** Asma Khalil, James M. N. Duffy, Helen Perry, Wessel Ganzevoort, Keith Reed, Ahmet A. Baschat, Jan Deprest, Eduardo Gratacos, Kurt Hecher, Liesbeth Lewi, Enrico Lopriore, Dick Oepkes, Aris Papageorghiou, Sanne J. Gordijn, On behalf of the International Collaboration to Harmonise Outcomes for Selective Fetal Growth Restriction (CHOOSE-FGR)

**Affiliations:** 1 Vascular Biology Research Centre, Molecular and Clinical Sciences Research Institute, St George’s University of London, Cranmer Terrace, London, SW17 0RE UK; 2 Fetal Medicine Unit, Department of Obstetrics and Gynaecology, St. George’s University Hospitals NHS Foundation Trust, Blackshaw Road, London, SW17 0QT UK; 3 Balliol College, University of Oxford, Broad Street, Oxford, OX1 3BJ UK; 4 Nuffield Department of Primary Care Health Sciences, University of Oxford, Radcliffe Primary Care Building, Radcliffe Observatory Quarter, Woodstock Road, Oxford, OX2 6GG UK; 5 Department of Obstetrics and Gynecology, Academic Medical Center Amsterdam, University of Amsterdam, Amsterdam, The Netherlands; 6 Twin and Multiple Births Association (TAMBA), The Manor House Manor Park, Church Hill, Aldershot, GU12 4JU UK; 7 The Johns Hopkins Center for Fetal Therapy, 600 North Wolfe, Nelson 228, Baltimore, MD 21287 USA; 8 Department of Obstetrics and Gynecology, University Hospitals of KU Leuven, Herestraat, 49 3000 Leuven, Belgium; 9 Barcelona Center for Maternal-Fetal and Neonatal Medicine, Hospital Clínic and Hospital Sant Joan de Deu, Universitat de Barcelona; Institut d’Investigacions Biomèdiques August Pi i Sunyer (IDIBAPS), and Centre for Biomedical Research on Rare Diseases (CIBER-ER), Barcelona, Spain; 10 Department of Obstetrics and Fetal Medicine, University Medical Center Hamburg-Eppendorf, Neues Klinikum, Gebäude O10 Martinistr. 52, 20246 Hamburg, Germany; 11 Division of Fetal Medicine, Department of Obstetrics, Leiden University Medical Center, K-06-35, P.O. Box 9600, 2300RC Leiden, The Netherlands; 12 Department of Obstetrics and Gynecology, University Medical Center Groningen, University of Groningen, Groningen, The Netherlands

**Keywords:** Selective fetal growth restriction, Selective intrauterine growth restriction, Core outcome set, Modified Delphi method, Modified Nominal Group Technique, Consensus development study

## Abstract

**Background:**

Selective fetal growth restriction in monochorionic twin pregnancies is associated with an increased risk of perinatal mortality and morbidity and represents a clinical dilemma. Interventions include expectant management with early preterm delivery if there are signs of fetal compromise, selective termination of the compromised twin, fetoscopic laser coagulation of the communicating placental vessels or termination of the whole pregnancy. Previous studies evaluating interventions have reported many different outcomes and outcome measures. Such variation makes comparing, contrasting, and combining results challenging, limiting ongoing research on this uncommon condition to inform clinical practice. We aim to produce, disseminate, and implement a core outcome set for selective fetal growth restriction research in monochorionic twin pregnancies.

**Methods:**

An international steering group, including professionals, researchers, and lay experts, has been established to oversee the development of this core outcome set. The methods have been guided by the Core Outcome Measures in Effectiveness Trials Initiative Handbook.

Potential core outcomes will be developed by undertaking a systematic review of studies evaluating interventions for selective fetal growth restriction in monochorionic twin pregnancies. Potential core outcomes will be entered into a three-round Delphi survey and key stakeholders including clinical professionals, researchers, and lay experts will be invited to participate. Repeated reflection and rescoring of individual outcomes should encourage group and individual stakeholder convergence towards consensus outcomes which will be entered into a modified Nominal Group Technique to finalize the core outcome set. Once core outcomes have been agreed, we will establish standardized definitions and recommend high-quality measurement instruments for each outcome.

**Discussion:**

The development, dissemination, and implementation of a core outcome set for selective fetal growth restriction should ensure that future research protocols select, collect, and report outcomes and outcome measures in a standardized manner. Data synthesis will be possible on a broad level and rigorous implementation should advance the quality of research studies and their effective use in order to guide clinical practice, improve patient care, maternal, short-term perinatal outcomes, and long-term neurodevelopmental outcomes.

**Trial registration:**

Core Outcome Measures in Effectiveness Trials (COMET) registration number: 998.

International Prospective Register of Systematic Reviews (PROSPERO) registration number: CRD42018092697. 18th April 2018.

**Electronic supplementary material:**

The online version of this article (10.1186/s13063-018-3153-y) contains supplementary material, which is available to authorized users.

## Background

Twin pregnancies complicated by selective fetal growth restriction (sFGR) are associated with increased perinatal mortality and morbidity [1]. This pathology affects 10–15% of monochorionic (MC) twin pregnancies and represents a management challenge due to the interdependence of twins connected via the placental vasculature [2–5]. One particular challenge in MC twin pregnancies complicated by sFGR is the risk of acute feto-fetal transfusion in the event of demise or hypoxia and vascular imbalance with profound hypotension in one twin causing death or neurological injury in the co-twin [6].

A classification according to the umbilical artery Doppler findings in the smaller twin was proposed in 2007, where three types with variable prognosis were identified [7]. Furthermore, we have recently published a consensus agreement on the diagnostic criteria of sFGR in twin pregnancies [8]. Although the overall perinatal survival in pregnancies affected by type 1 sFGR (with positive end-diastolic flow in the umbilical artery Doppler) is as high as 97%, the survival in sFGR with absent or reversed end-diastolic flow in the umbilical artery Doppler (types 2 and 3) is around 60 and 85%, respectively [3–5], with a high risk of intrauterine demise that may be particularly unpredictable in type 3 sFGR [1, 7]. Possible interventions include expectant management with early preterm delivery if there are signs of fetal compromise and active fetal intervention, which includes selective termination of the compromised twin, fetoscopic laser coagulation of the communicating placental vessels or termination of the whole pregnancy. Selective termination favors the outcome of the larger twin, while fetoscopic laser coagulation of the placental vessels can achieve survival of both twins in select cases at the cost of a higher risk of mortality and neurological complications in the larger co-twin [4, 5, 9, 10]. Clinicians and researchers face clinical uncertainty regarding the optimal management of sFGR [3–5]. Given the variation in outcome by classification, the optimal management is likely to vary according to the type and severity of sFGR, the distribution of placental tissue and the timing of onset.

Given the high potential for morbidity and mortality in these pregnancies, there is a need for robust guidance on the safest and most effective course of management. However, the large variation among the studies in the reported outcomes, makes it difficult to compare results, or combine the data from individual studies, limiting the potential of research to guide clinical practice. Core outcome sets are agreed, clearly defined minimum sets of outcomes that can be measured in a standardized manner and reported consistently [11]. Acknowledging that inconsistencies in outcome reporting can be disruptive to progress in our specialty, 78 editors of journals on women’s health came together to form a consortium to support the development, dissemination, and implementation of core outcome sets [12].

Our objective is to produce, disseminate, and implement a core outcome set for sFGR in MC twin pregnancies.

## Methods

**Fig. 1:**
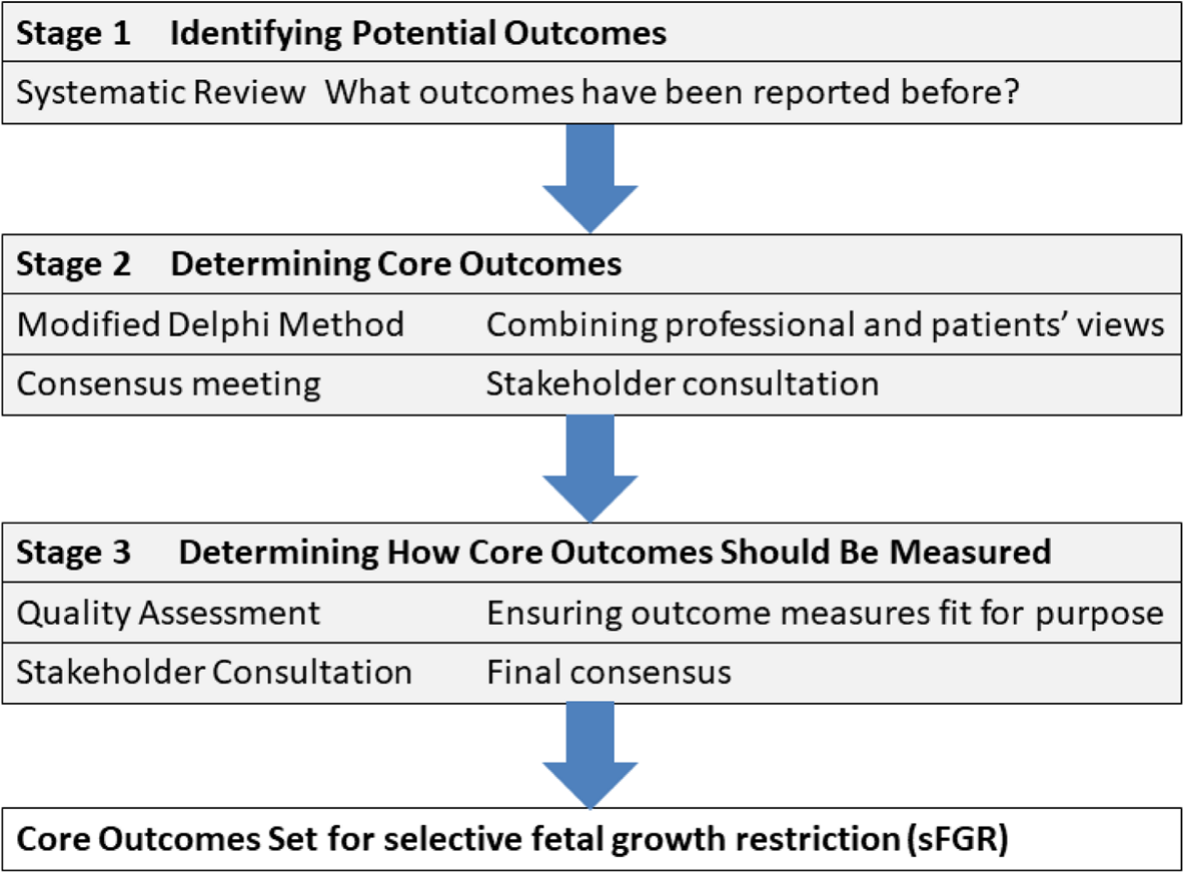
The stages of developing a core outcome set for selective fetal growth restriction

The methods have been informed by the Core Outcome Measures in Effectiveness Trials (COMET) Initiative Handbook and other core outcome set development studies relevant to women’s and newborn health, including twin-to-twin transfusion syndrome, pre-eclampsia, endometriosis, termination of pregnancy, and neonatal medicine [11, 13–17]. This study protocol is reported in accordance with Standard Protocol Items: Recommendations for Interventional Trials (SPIRIT) Guidelines (Additional file 1). The stages of the development of the core outcome set are shown in Fig. 1.

### Prospective registration

This study has been registered with the International Prospective Register of Systematic Reviews (PROSPERO) (registration number: CRD42018092697 and The Core Outcome Measures in Effectiveness Trials (COMET) Initiative (registration number: 998).

### Steering Group

An international steering group, including professionals, researchers, and women with lived experience of sFGR, has been formed to guide the development of this core outcome set. The Steering Group members were approached and invited based on their expertise as clinicians and researchers in sFGR. Patient representatives were approached as interested individuals who had previously contacted The Twin and Multiple Births Association (TAMBA). The Steering Group has been established to make decisions regarding the study’s methods; for example, determining the scope of the core outcome set, selecting appropriate consensus methods, and developing the recruitment strategy. While the Steering Group will oversee the process, further participants will be involved in the consensus-forming process and anyone, anywhere is welcome to participate.

### Scope of the core outcome set

This core outcome set will apply to all therapeutic interventions for sFGR. This will not be limited by the type of intervention, the setting in which administered, or the gestation at which provided. Selective fetal growth restriction will be defined according to the recently published consensus agreement on the diagnostic criteria of sFGR in twin pregnancies; the solitary finding of an estimated fetal weight below the third centile in one of the twins or at least two out of four of the following: (1) an estimated fetal weight below the 10th centile in one of the twins, (2) an abdominal circumference below the 10th centile in one of the twins, (3) an estimated fetal weight discordance ≥ 25%, and (4) umbilical artery Doppler pulsatility index > 95th centile in the smaller twin [8]. However, as this consensus was only recently published, studies that use other definitions, the commonest is one twin with an estimated fetal weight less than the 10th centile, will be included. We are not seeking to reach consensus on the standardization of other aspects of study design or the definition or staging of sFGR.

### Identifying potential core outcomes

We will conduct a systematic review to identify potential core outcomes. The systematic review methods will be informed by published systematic reviews identifying potential core outcomes [18–23]. We will search the Cochrane Central Register of Controlled Trials (CENTRAL), EMBASE, and MEDLINE from inception to January 2018 to identify all trials and observational studies reporting outcomes evaluating interventions for sFGR using defined Medical Subject Headings (MeSH) descriptor terms including sFGR and selective intrauterine growth restriction. We will search unpublished gray literature and ongoing trials and observational studies using the same terms. The conduct of the systematic review will adhere to standard systematic review methods [24]. The population includes MC twin pregnancies complicated by sFGR. The interventions include any intervention used for the treatment of sFGR. The comparator will include any comparator treatment used for the interventions of sFGR. Possible interventions include alternative treatment, standard care, a placebo treatment or no treatment. The outcomes will include all outcomes reported in the included sFGR studies. We will include all randomized controlled trials, quasi-randomized trials, controlled before and after studies, interrupted time series, and observational studies that report an outcome following any intervention for sFGR. We will exclude case reports, small case series, editorials, and review articles. No data or language limits will be applied.

All studies identified in the search will be screened using the title and abstract. The full-text article will be reviewed of all studies meeting the inclusion criteria and those where this cannot be determined from the abstract alone. Identified studies will be reviewed by two reviewers and any discrepancies resolved by discussion with a third reviewer. The studies will be assessed using a purposively developed Data Extraction Form to collect the following information: year of publication, study design, sample size, intervention undertaken, outcomes, and outcome measures.

We will report the systematic review with reference to the Preferred Reporting Items for Systematic Reviews and Meta-analyses (PRISMA) Statement for reporting systematic reviews and meta-analyses of studies that evaluate health care interventions [25]. All identified outcomes will be entered into an outcome inventory and organized into the following categories: survival outcomes, fetal outcomes, short-term neonatal outcomes, long-term neurodevelopmental outcomes (including behavioral outcomes and quality of life), obstetric outcomes and surgical/operator outcomes. These outcomes will be reviewed and discussed by the Steering Group with particular emphasis on reducing duplication of outcomes caused by varying terminologies and grouping very similar outcomes together in order to make the final outcome inventory clear and succinct. Following agreement, the inventory will be entered into the modified Delphi method. The wording of the outcomes will be decided in collaboration with the patient representatives (TAMBA).

### Determining core outcomes

The core outcomes will be determined by using a modified Delphi method. The Delphi method is an established tool for achieving a convergence of opinion on a particular subject by gathering data from respondents with expert knowledge of that particular subject. It allows consensus building by using a series of questionnaires to extract opinion from participants. Web-based Delphi tools facilitate international data collection and are largely considered acceptable to the user [26, 27]. All categories of stakeholder, including health professionals, researchers and people with lived experience of or expertise in sFGR will be invited to take part. By asking participants to specify the stakeholder group that they most identify with, we will be able to interpret the results of the survey both as a whole and by participant background, and to identify any differences between the groups. The recruitment strategy has been designed to ensure that people with assorted experiences of sFGR from diverse demographic backgrounds and geographical locations can be recruited. We will ask members of an expert panel who have participated in the core outcome set on twin-to-twin transfusion syndrome and those who participated in the development of a consensus on the diagnostic criteria of sFGR. Recruitment will be facilitated by national and international patient organizations, including TAMBA, and national and international professional organizations, including the Royal College of Obstetricians and Gynaecologists (RCOG), the International Society of Ultrasound in Obstetrics and Gynaecology (ISUOG), and the International Society for Twin Studies (ISTS), advertising the study within their newsletters, online forums, and social media feeds. Potential participants will be able to register their interest online and will be sent Delphi survey instructions written in plain language. When participants register to complete the survey, they will complete a questionnaire recording demographic details; for example, age, ethnic group, and country, and information pertaining to their experiences or expertise of sFGR. We aim to recruit 18 participants for each stakeholder group. Participants who fail to complete the Delphi survey will be asked about their reasons for withdrawal. Only core members of the Steering Group will have access to the study dataset.

### Round 1

All participants will be invited to register with the online survey and will be allocated a unique identifier to enable anonymization of their responses. They will be asked to score individual outcomes on a 9-point Likert scale anchored between one (labeled “of limited importance for making a decision”) and 9 (labeled “critical for making a decision”). This scale was devised by the Grading of Recommendations Assessment, Development and Evaluation (GRADE) Working Group to facilitate the ranking of outcomes according to their importance and has been adopted widely by core outcome set developers [28]. There will be an opportunity for participants to suggest new outcomes and these will be considered by the Steering Group for inclusion in the second-round survey.

### Round 2

All outcomes will be carried forward into the second round and any additional outcomes suggested by participants will be included. Participants will receive their own scores and stakeholder group feedback for each round-1 outcome. Participants will be invited to reflect and rescore individual outcomes.

### Round 3

All outcomes will be carried forward into the third round. Participants will receive their own scores and stakeholder group feedback for each round-2 outcome. Participants will be invited to reflect and rescore individual outcomes. A standardized definition will be applied to this round’s results enabling consensus outcomes to be identified. Consensus outcomes will be defined as outcomes achieving a median score of 8 across all three stakeholder groups.

### Stakeholder meeting: modified Nominal Group Technique

The results of the Delphi survey will be discussed in a consensus development meeting including professionals, researchers and people with lived experience of sFGR. The round-3 results will be reviewed and all consensus outcomes will be considered. Participants will be able to discuss any other outcomes upon request. The objective of the consensus development meeting will be to develop a final core outcome set for sFGR.

### Determining core outcome measures

Once the core outcome set has been agreed and established it will be necessary to determine how it should be defined or measured. Potential definitions and measurement instruments will be inventoried across formal definition development initiatives, national and international guidelines, Cochrane systematic reviews and published studies. Potential definitions will be entered into a consensus development workshop including professionals, researchers and people with lived experience of sFGR. The objective of the consensus workshop will be to identify definitions for individual core outcomes. Potential measurement instruments will be quality assessed using the Core Outcome Measures in Effectiveness Trials (COMET) and the Consensus-Based Standards for the Selection of Health Measurement Instruments (COSMIN) Initiative quality assessment framework [29].

### Dissemination and implementation

The Steering Group will aim to disseminate the core outcome set as widely and effectively as possible. We will aim to describe the core outcome set through publication in a relevant journal as well as presenting to our peers at meetings. The Core Outcomes in Women’s and Newborn’s Health (CROWN) Initiative is endorsed by the International Federation of Gynecology and Obstetrics (FIGO), the Royal College of Obstetricians and Gynaecologists (RCOG), and the Royal Australian and New Zealand College of Obstetricians and Gynaecologists (RANZCOG). TAMBA will use its publicity channels to further share the core outcome set to both health care professionals and patients. With the CROWN Initiative now supported by over 80 journals, researchers will have more obligation to engage with the core outcome set when it comes to planning their studies. We will also engage with the relevant Cochrane Review Groups, clinical guideline developers, research funders, trial registries and regulators such as research ethics committees. We plan to use a theoretically informed framework when implementing dissemination [30]. The methods of dissemination we are planning to use are peer-reviewed publications in open-access journals, presentations at scientific meetings, posters, events, newsletters, press release, and podcasts. We will report the core outcome set in line with the COS-STAR Statement [31].

## Discussion

The development and implementation of core outcome sets is likely to be very beneficial to the design and reporting of clinical studies, systematic reviews, and clinical guideline. This should ultimately improve clinical care and patients’ experience. The importance of such an initiative has been acknowledged by a number of key national and international organizations.

### Improving the selection of the outcome of clinical studies

The Standard Protocol Items: Recommendations for Interventional Trials (SPIRIT) Statement, supported by funders of health research, such as the National Institute of Health Research (NIHR), recommends the use of core outcome sets. The use of standard core outcome sets would enhance comparability of clinical trials and facilitate the conduct of prospective meta-analyses using individual patient data.

### Facilitating the evidence synthesis and reporting of clinical studies

The Core Outcomes in Women’s and Newborn’s Health (CROWN) Initiative, supported by 78 specialty journals, including the Cochrane Pregnancy and Childbirth Group, has resolved to implement core outcome sets. These journals would expect authors to report the study results for the core outcomes and draw their conclusions based on these outcomes rather than non-core or surrogate outcomes.

### Enhancing the ability to develop robust clinical guidelines

The National Institute for Health and Care Excellence (NICE) supports the use of core outcome sets during evidence scoping and synthesis [32, 33]. The NICE methodology of assessment of the quality of the evidence takes into account whether the data of interest were reported as a core, non-core or surrogate outcome. This initiative to improve the quality and consistency of outcomes investigated and reported by researchers can in turn lead to the development of guidelines based on clearer and stronger evidence to help all clinicians offer the best interventions for their patients.

### Developing a network which can support an international collaboration

The team of involved key stakeholders has the potential to set up an international network, which could be a potent vehicle for the development of international guidelines and registries and setting research priorities for sFGR. In the context of sFGR, this could potentially have a profound impact on morbidity and mortality rates in the long term.

## Trial status

At the time of manuscript submission, the systematic review process has commenced and strategic planning for the Delphi method consensus-building exercise is underway. This is the first version of the protocol (18th April 2018).

## Additional files

Additional file 1: Populated Standard Protocol Items: Recommendations for Interventional Trials (SPIRIT) Checklist. (DOC 120 kb)

Additional file 2: The National Research Ethics Service (NRES) has advised that ethical approval is not required. (PDF 164kb)

## Abbreviations

CENTRAL: Cochrane Central Register of Controlled Trials
COMET: Core Outcome Measures in Effectiveness Trials Initiative
COMIS: Core Outcome Measurement Instrument Selection project
COS-STAR: Core Outcome Set Standards for Reporting
CROWN: Core Outcomes in Women’s and Newborn’s Health Initiative
GRADE: Grading of Recommendations Assessment, Development and Evaluation Working Group
ISUOG: International Society of Ultrasound in Obstetrics and Gynaecology
MC: Monochorionic
MeSH: Medical Subject Headings
NICE: National Institute for Health and Care Excellence
NRES: National Research and Ethics Service
PRISMA: Preferred Reporting Items for Systematic Reviews and Meta-analyses
PROSPERO: International Prospective Register of Systematic Reviews
RANZCOG: The Royal Australian and New Zealand College of Obstetricians and Gynaecologists
RCOG: Royal College of Obstetricians and Gynaecologists
sFGR: Selective fetal growth restriction
SPIRIT: Standard Protocol Items : Recommendations for Interventional Trials Statement
